# Blended Polymer Dry Electrodes for Reliable Electrocardiogram and Electromyogram Measurements and Their Eco-Friendly Disposal Led by Degradability in Hot Water

**DOI:** 10.3390/polym14132586

**Published:** 2022-06-26

**Authors:** Dong Hyun Lee, Eun Kwang Lee, Chae Hyun Kim, Hyung Joong Yun, Young-Joon Kim, Hocheon Yoo

**Affiliations:** 1 Department of Electronic Engineering, Gachon University, 1342 Seongnam-daero, Seongnam 13120, Korea; danny99hodam@gachon.ac.kr (D.H.L.); chrabbit27@naver.com (C.H.K.); 2 Department of Chemical Engineering, Pukyong National University (PKNU), Busan 48513, Korea; eklee@pknu.ac.kr; 3 Advance Nano Research Group, Korea Basic Science Institute (KBSI), Daejeon 34126, Korea; hjyun@kbsi.re.kr

**Keywords:** electrocardiography, electromyography, PEDOT:PSS, degradability, polymer electrode

## Abstract

To increase the human lifespan, healthcare monitoring devices that diagnose diseases and check body conditions have attracted considerable interest. Commercial AgCl-based wet electrodes with the advantages of high conductivity and strong adaptability to human skin are considered the most frequently used electrode material for healthcare monitoring. However, commercial AgCl-based wet electrodes, when exposed for a long period, cause an evaporation of organic solvents, which could reduce the signal-to-noise ratio of biosignals and stimulate human skin. In this context, we demonstrate a dry electrode for a poly(3,4-ethylenedioxythiophene):poly(styrenesulfonate) (PEDOT:PSS)-based blended polymer electrode using a combination of PEDOT:PSS, waterborne polyurethane (WPU) and ethylene glycol (EG) that could be reused for a long period of time to detect electrocardiography (ECG) and electromyography (EMG). Both ECG and EMG are reliably detected by the wireless real-time monitoring system. In particular, the proposed dry electrode detects biosignals without deterioration for over 2 weeks. Additionally, a double layer of a polyimide (PI) substrate and fluorinated polymer CYTOP induces the strong waterproof characteristics of external liquids for the proposed dry electrodes, having a low surface energy of 14.49 mN/m. In addition, the proposed electrode has excellent degradability in water; it dissolves in hot water at 60 °C.

## 1. Introduction

To ensure the world’s aging population has the right to individual life, neurological diseases, cardiovascular diseases, etc. are becoming notable social issues. As a result, many studies have been carried out to develop high-performance electrocardiograph (ECG) and electromyograph (EMG) sensors that can efficiently obtain biosignals from the surface of the human body. Especially, a non-invasive biopotential electrode is well known for its key contribution as a component of a biopotential acquisition system from a human being due to its fast measurement setup on the surface of the human body without leaving a scar. Robust and reliable biosignal acquisition is strongly dependent on the characteristic of biopotential electrodes, such as adhesion, wet-degradability, conductivity and micro-/macroscopic morphology.

So far, much work on the development of biopotential electrodes has been carried out in terms of material properties and mechanical flexibility to record biosignals. Commercially, wet-type electrodes (typically, Ag/AgCl) have been widely utilized because of their high conductivity and conformability to human skin [1,2,3]. However, electrolytes of the wet-type electrodes are diffused into the subcutaneous area of the human body, and organic solvents of the wet-type electrodes evaporated over a long period of time. Thus, this would cause skin irritation and significant decays of the signal-to-noise ratio of biosignals, respectively. Instead, the fabrication of dry-type electrodes was intensively investigated using a thin metal [4,5,6,7], a carbon nanotube (CNT) [8,9,10,11], polymer–metal particle composites [12], graphene [13,14,15,16,17] and conductive polymers, such as poly(3,4-ethylenedioxythiophene):poly(styrenesulfonate) (PEDOT:PSS) [18,19,20].

Major technological issues in the fabrication of biopotential electrodes are mechanical robustness and reliability for long-term biosignal recording. A previously reported study fabricated organogel-based strain-insensitive conductors using PEDOT:PSS, polyacrylamide (AAm) and ethylene glycol (EG) [18]. Excessive ions and impurities in PEDOT:PSS were eliminated via dialysis. In addition, EG, which has a high boiling point (197 °C), scarcely evaporated at room temperature, so organogel-based PEDOT:PSS maintained the compositional ratio over a long time. Biocompatible PEDOT:PSS electrode was fabricated by blending a waterborne polyurethane (WPU) and D-sorbitol, which is a food additive used in various fields such as cosmetic lotions, creams, toothpastes and food additives [20]. The fabricated dry-type PEDOT:PSS electrodes exhibited excellent electrical conductivity, strong adhesion properties and long-term stability. Complex microstructured and macropillar-shaped biopotential electrodes have been reported by a research group of ETH Zurich, which exhibited low electrical impedance [21]. The complex microstructured electrodes and macropillar-shaped electrodes were fabricated using multiple photolithography and a stencil printing technique, respectively. However, the fabrication of the biopotential electrodes involved multiple steps of the lithographic process and could suffer from the reduction in the fabrication process yield. From these points of view, human-friendly and environmentally friendly biopotential electrodes are highly required, with simple yet robust film formation using conductive polymers.

PEDOT:PSS, a representative high-conductivity polymer, is a polymer mixture composed of conductive PEDOT and insulating PSS. PEDOT:PSS has attracted attention over the decades due to high transmittance in the visible light region and solution processability. PEDOT:PSS can be applied to various electronic devices and display fields, such as wearable devices, transparent electrodes and solar cells [22,23,24]. Recently, research has been reported to improve the conductivity, thermoelectric characteristics and mechanical flexibility of PEDOT:PSS. Ionic liquids such as butyl-3-methylimidazolium, tetrafluoroborate and 2-methylimidazolium hydrogen sulfate improve the conductivity of PEDOT:PSS [25,26]. In addition, deep eutectic solvents (DES), which are biocompatible, and biodegradable solvents increased the thermoelectric and sensing characteristics of PEDOT:PSS [27,28]. Lastly, the addition of elastomer to PEDOT:PSS leads to high mechanical characteristics [29]. The PEDOT:PSS composite, with improved conductivity, sensing and mechanical characteristics, is a strong potential candidate for use as an electrophysiological epidermal electrode.

In this light, we present waterproof dry-type PEDOT:PSS (WPD) electrodes covered with a commercially available fluorinated polymer (CYTOP), WPU and polyethylenimine (PEI) on a polyimide (PI) substrate to measure human electrocardiography (ECG) and electromyography (EMG). Due to the doubled cover of CYTOP on the electrode structure, a powerful waterproof characteristic was realized on the ECG and EMG electrodes. The waterproof dry electrode sensed biosignals without deterioration over 2 weeks. Moreover, the material constituting the dry electrode had an excellent eco-friendly degradable characteristic to minimize the production of electronic waste. Our proposed PEDOT:PSS-based dry electrode demonstrated a stable ECG and EMG signal recording wirelessly with real-time monitoring.

## 2. Materials and Methods

The PI substrate that acted as a framework to form the proposed electrode was cut to a certain size (2 cm × 2 cm). The standardized PI substrate was sonicated in acetone and isopropyl alcohol (IPA) for 10 min sequentially and then dried with nitrogen gas (99.99%). After all the residue was removed in an oven at 95 °C for 5 min, the CYTOP solution was diluted 1:5 by CYTOP solvent (CT-SOLV180) and stirred for 6 h. The CYTOP solution with 0.5 mL was coated on the standardized PI substrate at 3000 rpm for 30 s and then annealed with a hotplate at 100 °C for 20 min, and 150 °C for 1 h sequentially in order to remove the CYTOP solvent. A total of 0.5 mL of the PEI solution (Sigma-Aldrich, St Louis, MO, USA) was coated at 3000 rpm for 30 s in the opposite layer of the PI substrate, in which the CYTOP was not coated to form an adhesion layer for selective molding and firm fixation of the PWE solution. The PWE solution was prepared by mixing a PEDOT:PSS solution (Heraeus, Hanau, Germany), WPU (Sigma-Aldrich, St Louis, MO, USA) and ethylene glycol (Sigma-Aldrich, St Louis, MO, USA) overnight (mixing ratio, 6:1:2). The WPU solution was prepared by stirring overnight at a ratio of 10:1, using deionized water as a solvent. The prepared PWE solution was drop-cast on the coated PEI layer and then, a 110 °C annealing process was performed in an oven. The drop-casting process of the PWE solution and annealing process using the oven was repeated 4 times (total of 2500 mL: 500 mL, 500 mL, 750 mL, 750 mL).

To investigate the chemical structure and the change in the energy level of WPD electrodes, the X-ray photoelectron spectroscopy (XPS) and ultraviolet photoelectron spectroscopy (UPS, AXIS Supra, Kratos, Manchester, UK) measurements were performed and using a monochromatic Al Kα (hv = 1486.6 eV) source. The morphological properties of the WPD electrodes were characterized by scanning electron microscopy (SEM, S-4700, Hitachi, Tokyo, Japan) and atomic force microscopy (AFM, Park NX10, Park systems, Suwon, South Korea). The image size of AFM was 10 µm × 10 µm, and the resolution was 0.05 nm. The surface energy was investigated by contact angle measurements (DSA100, KRUSS, Hamburg, Germany) and calculated with KRUSS advanced software.

## 3. Results and Discussion

**Figure 1:**
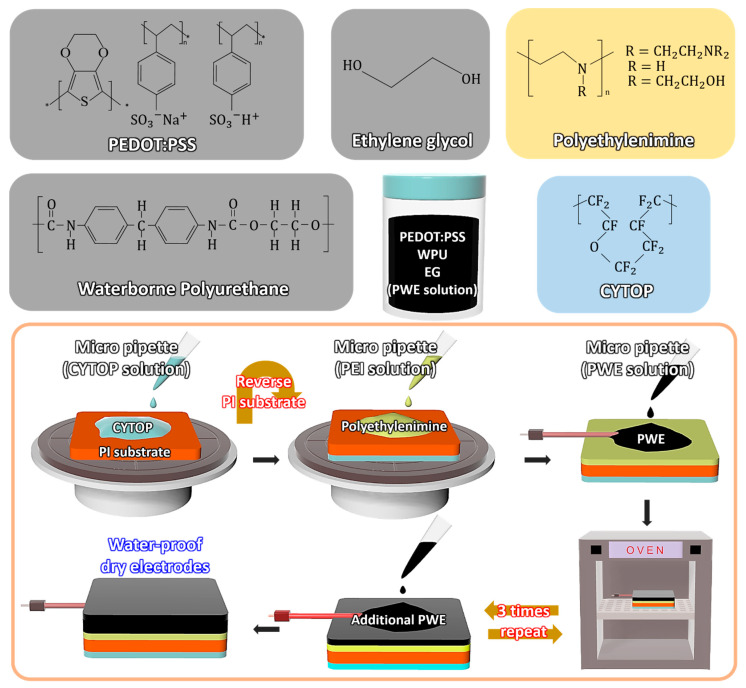
The 3D illustration of the waterproof dry-type PEDOT:PSS (WPD) electrode fabrication process and chemical structure of constituent materials.

Figure 1 shows the fabrication process of the proposed waterproof dry electrode and the chemical structures of the materials. The proposed electrode which detects the human biopotential signals such as ECG and EMG was composed of three polymers: PEDOT:PSS [30,31], WPU [32,33] and EG [34]. PEDOT:PSS is a polymer with high conductivity and was used as the main active material of the proposed electrode. In addition, PEDOT:PSS, which has high transmittance in visible light regions and solution processability, has attracted attention in fields such as wearable devices, transparent electrodes and solar cells. However, PEDOT:PSS film is not suitable for reliable contact with moving human skin due to its relatively low physical durability and flexibility. The added WPU provides elasticity and flexibility characteristics to PEDOT:PSS films to minimize the damage to the electrode due to the movement of the human body. In addition, EG provides additional conductivity to the PEDOT:PSS films, allowing the WPD electrode to reliably detect ECG and EMG biopotential signals. The PEI plays the role of the adhesive layer between the blended polymer electrode solution and the PI substrate. Moreover, the hydrophobic CYTOP layer coated on the PI substrate protects the WPD electrode from external liquids. The PI substrate acts as the framework for the WPD electrode and leaves an additional waterproof effect. A detailed description of the fabrication process of the waterproof dry electrode is presented in the Section 2. 

**Figure 2:**
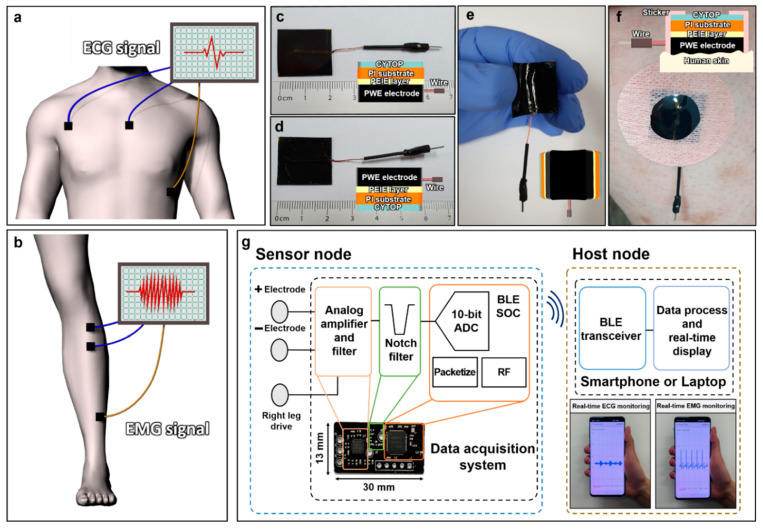
The ECG and EMG biopotential signal detecting system, and the wireless real-time monitoring system. The 3D illustration of 2-electrode systems and 3-electrode systems for detecting human ECG and EMG biopotential signals. Blue electrode: positive and negative electrodes, yellow electrode: reference electrode. (**a**) The position of WPD electrodes attached to a human chest to detect the ECG biopotential signals; (**b**) the position of WPD electrodes attached to a human right leg to detect the EMG biopotential signals; the photography of the fabricated WPD electrodes; (**c**) the front side of the WPD electrodes; (**d**) the back side of the WPD electrodes; (**e**) the flexibility of the WPD electrodes; (**f**) the WPD electrodes attached to a real human body; (**g**) the block diagram of a real-time monitoring system.

The position of the WPD electrodes attached to the human body for detecting the ECG and EMG biopotential signals and the shape of the waveform are shown in the 3D illustration (Figure 2a,b). There were two-electrode and three-electrode systems for detecting ECG and EMG biopotential signals, respectively. The two-electrode system was composed of positive and negative electrodes, whereas the three-electrode system added a reference electrode to set the biopotential standard. It is noted that a blue-wired electrode indicates positive and negative electrodes, while a yellow electrode indicates a reference electrode. Figure 2c and d show the front side and back side photography of the fabricated WPD electrode, respectively. In addition, the WPD electrode with flexible and elastic characteristics by the PI substrate is shown (Figure 2e). Figure 2f shows the WPD electrode was attached to real human skin using a medical sticker to detect the biopotential signals. 

Figure 2g is a block diagram of a real-time monitoring system to obtain ECG and EMG biopotential signals. The system was divided into two sub-systems: the sensor node and the host node. In the sensor node, there was a 180 kΩ resistor placed between the WPD electrode and the amplifier to ensure that the current flow never exceeded 10 μA. The analog amplifier and filter block conditioned the biosignal acquired from the WPD electrode with a voltage gain of 60 dB (AD8232, Analog Devices). An instrumentation amplifier initially amplified the signal from the WPD electrodes and attenuated common-mode signals. An active filter conditioned the signal with a second-order high-pass and a low-pass active filter to eliminate unnecessary motion artifacts and high-frequency noise. The active filter was designed with a passband from 0.34 Hz to 41 Hz and 40.17 Hz to 727 Hz for ECG and EMG, respectively. A right leg drive circuit was used to further improve the common-mode rejection. To eliminate powerline noise caused by general consumer electronics, a Twin-T notch filter block was implemented. The biosignals were then sampled at 12 bits with a sampling rate of 7 kSps using the Analog-to-Digital Converter (ADC) embedded in the Bluetooth low-energy system-on-chips (BLE SoC, nRF52832, Nordic Semiconductor). The sampled data were collected in a buffer and transmitted from a burst mode to the host node every 24 ms. This sensor node sub-system was manufactured in a printed circuit board (PCB) with a size of 13 mm x 30 mm. The wirelessly transmitted data were received by the host node, which could either be a smartphone or a personal computer, where the data were displayed in real time by a custom-made software application.

**Figure 3:**
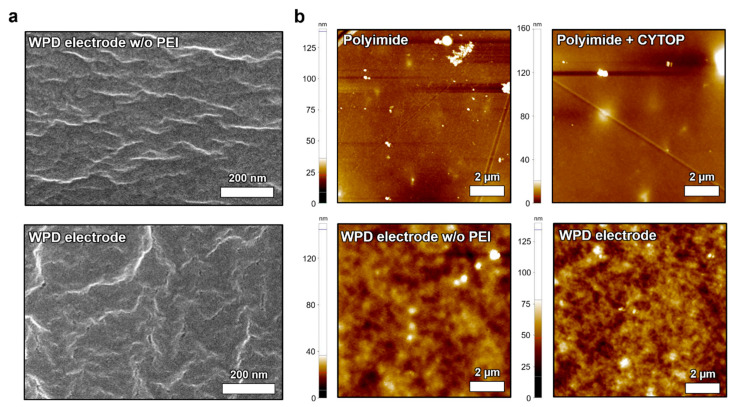
The morphological analysis of the WPD electrodes. The SEM surface image of (**a**) the WPD electrode layer without PEI and WPD electrode layer; the AFM surface image of (**b**) the PI substrate, CYTOP coated PI substrate, WPD electrode layer without PEI and WPD electrode layer.

SEM measurement was used for the analysis of WPD electrodes (Figure 3a). SEM images of the WPD electrode layer without PEI and the WPD electrode layer were captured. The wrinkles were observed on the surfaces of both dry electrodes fabricated using the drop-casting process. AFM measurement was conducted to analyze the surface properties of the proposed WPD electrode layer with or without the PEI layer and, additionally, the presence or absence of CYTOP on the PI substrate was confirmed (Figure 3b). The roughness of the PI substrate and the PI substrate on CYTOP was 6.96 nm and 7.13 nm, respectively. In addition, the roughness of the WPD electrode layer without PEI was 7.45 nm, whereas it increased to 15.44 nm when the PEI layer was added. The surface was not smooth, due to the two types of WPD electrodes produced via drop-casting. Lastly, the presence of the PEI layer increased the contact surface area with human skin with additional roughness, allowing for stable ECG and EMG biopotential signal detection [35].

**Figure 4:**
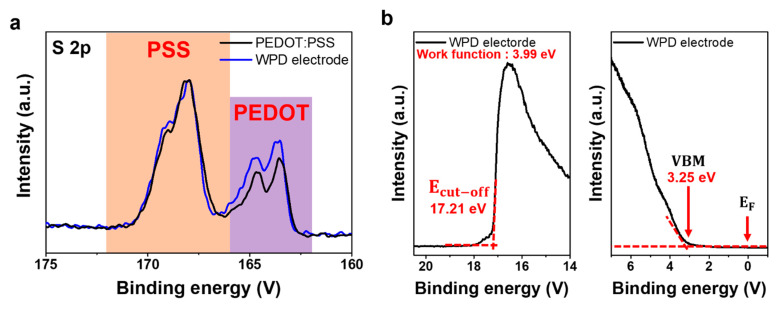
(**a**) The XPS analysis of the PEDOT:PSS and the WPD electrode; (**b**) the UPS analysis of the optimized WPD electrode.

XPS was performed to shed light on the chemical structure of the WPD electrodes. Figure 4a shows the S 2p spectra of PEDOT:PSS and WPD electrodes. The PEDOT chain peak occurred in the range of binding energy of 162 to 166 eV and the PSS chain peak occurred in the range of binding energy of 166 to 172 eV (Figure 4b) [36,37]. The PEDOT chain peak intensity of WPD increased more than PEDOT:PSS, which indicated an improvement in conductivity [38]. Sheet resistance measurement was performed to investigate the conductivity of PEDOT:PSS and WPD electrodes (Supplementary Figure S1). The measured sheet resistance of PEDOT:PSS and WPD electrodes was equal to 10.67 Ω and 1.158 Ω, respectively. The sheet resistance of the WPD electrode was improved by EG. The EG enhanced the conductivity of PEDOT:PSS by removing the insulating PSS chains from PEDOT:PSS. Figure 4b shows the UPS spectra of the WPD electrode with the optimized composition ratio. The valence band maximum (VBM) and the cut-off of the WPD electrode were plotted as 17.21 eV and 3.25 eV, respectively. As a result, the WPD electrode had a work function of 3.99 eV, which was reduced by 0.85 eV, compared to the work function of the pure PEDOT:PSS of 4.84 eV (Supplementary Figure S2). The optical bandgaps of PEDOT:PSS and WPD electrodes were calculated using the Tauc plot method (Supplementary Figure S3). The optical bandgaps of the WPD electrode and the PEDOT:PSS electrode were 5.13 eV and 5.12 eV, respectively. The 0.01 eV energy bandgap change between the PEDOT:PSS electrode and the WPD electrode was negligible. When WPU and EG were added to PEDOT:PSS to fabricate WPD electrodes, the Fermi level increased by 0.85 eV, while the energy band was maintained unchanged. 

**Figure 5:**
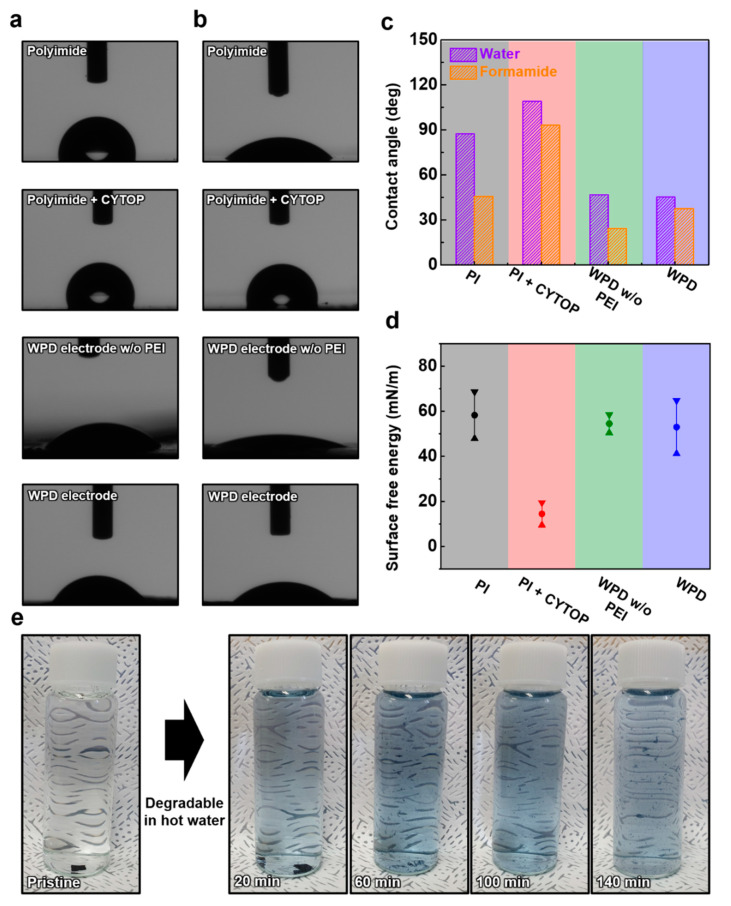
The analysis of contact angle. (**a**) The image of contact angle with deionized water; (**b**) the image of contact angle with formamide; (**c**) the histogram graph of contact angle with deionized water and formamide; (**d**) the graph of surface energy; (**e**) photography of degraded the WPD electrode over time.

The contact angle analysis was performed for the hydrophobic characteristics of a CYTOP-coated PI substrate and the investigation of the surface energy with the WPD electrode layer. The deionized water (DI water) and formamide were used to measure the contact angle and surface energy (Figure 5a,b). Figure 5c shows the histogram graph of the contact angle with deionized water and formamide. When CYTOP was coated on the PI substrate, the contact angle increased from 87.35° to 109.09° in DI water and also increased from 45.73° to 93.16° in formamide. Additionally, the contact angle characteristics of the WPD electrode without the PEI layer and the WPD electrode were analyzed. The contact angle between the WPD electrode without the PEI layer and the WPD electrode by DI water changed from 46.86° to 45.22°, and the difference of 1.64 degrees was negligible. On the other hand, the contact angle at formamide changed from 24.37° to 37.47°, and the difference was 13.1°. Additionally, the surface energy of four types of samples (PI substrate, CYTOP-coated PI substrate, WPD electrode without PEI layer and WPD electrode) was analyzed. The surface energy of the PI substrate and the CYTOP-coated PI substrate was 58.22 mN/m and 14.49 mN/m, respectively. On the other hand, the surface energy of the WPD electrode without the PEI layer and the WPD electrode was 58.53 mN/m and 52.96 mN/m, respectively. As a result, the PI substrate that prevented the physical penetration of external liquids and the CYTOP with strong hydrophobicity prevented the decomposition of the WPD electrode by liquid. [39,40]. To demonstrate the eco-friendly disposal of the proposed WPD electrodes, a degradability test was performed. Figure 5e shows photography of the WPD electrode immersed in DI water at 60 °C. The WPD electrode that was ultrasonicated had totally degraded, leaving tiny fragments after 140 min. This shows that PEDOT, WPU and EG composing the WPD electrode all have the characteristics of being degradable by water, and that degradability proceeds easily with external stimuli. 

Additionally, degradability tests of the WPD electrodes immersed in cold water were performed (Supplementary Figure S4). The WPD electrodes immersed in cold water (22 °C) were degradable in 600 min, with the exception of small fragments. The degradable rate of WPD electrodes in cold water was about 460 min slower than in hot water. The observed result indicates that hot water at 60 °C activates the chemical reaction of the WPD electrode, causing it to degrade quicker [41].

**Figure 6:**
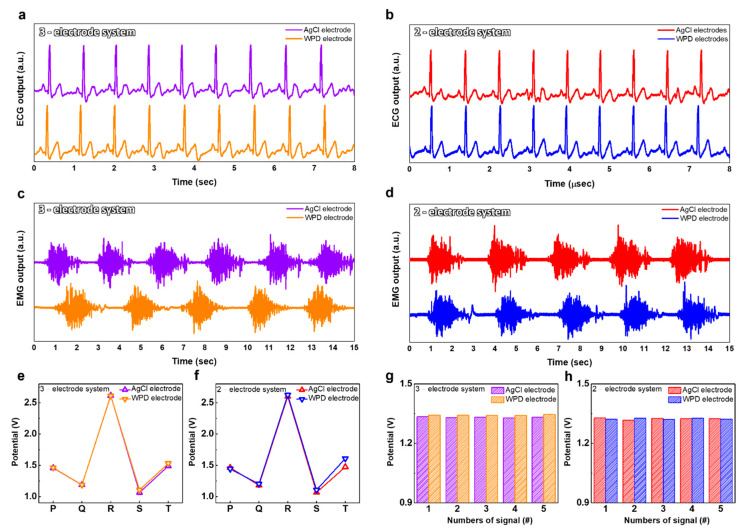
The biopotential signals of ECG and EMG with the WPD electrodes and commercial AgCl-based electrodes. (**a**) The ECG biopotential signals in 3-electrode system; (**b**) the ECG biopotential signals in 2-electrode system; (**c**) the EMG biopotential signals in 3-electrode system; (**d**) the EMG biopotential signals in 2-electrode system; (**e**) the PQRST peak in 3-electrode system; (**f**) the PQRST peak in 2-electrode system; (**g**) the quantified EMG biopotential signals in 3-electrode system; (**h**) The quantified EMG biopotential signals in 2-electrode system.

The ECG and EMG biopotential signals were confirmed using the proposed WPD electrode. The ECG and EMG biopotential signals were received from the chest and left leg, respectively. Additionally, the medical sticker was used to immobilize the electrodes with human skin. We compared it with a commercial AgCl-based electrode to prove the superiority of the WPD electrode in the detection of ECG and EMG biopotential signals. The ECG and EMG biopotential signals were measured using both the three-electrode system and the two-electrode system (Figure 6a–d). The ECG biopotential signal measured with the WPD electrodes clearly defined the peaks of the PQRST. The ECG could be identified by the repetitive P wave, QRS complex and T wave. The P wave and QRS complex were generated by atrial depolarization and ventricular depolarization, respectively. In addition, the T wave was caused by the repolarization of the ventricles. [42] Additionally, the EMG biopotential signals, which represent the movement of human muscle, were defined and gathered using the miniaturized monitoring system. In addition, we also plotted the PQRST peak of the ECG waveform measured with the WPD electrodes, compared to a commercial AgCl-based electrode used to investigate the ability to discriminate the waveform of the ECG measured (Figure 6e,f). In the three-electrode system, the change in the PQRST biopotential signal of ECG detected with WPD electrodes averaged 16 mV. In addition, the biopotential of the T peak, detected with the two-electrode system using the WPD electrode, was measured to be more than 130 mV higher than the commercial AgCl-based electrode, which proves that the ECG biopotential signals are measured more clearly with the WPD electrode. Figure 6f,g show the quantified EMG biopotential signals that plotted the five waveforms. The EMG biopotential signal change in the three-electrode system was measured to 10 mV, whereas the two-electrode system was measured to 6 mV. As a result, the WPD electrode and the commercial AgCl-based electrode show similar capabilities for detecting ECG and EMG biopotential signals. Note that the voltage readings were based on the amplification and filtering process of the sensor node.

**Figure 7:**
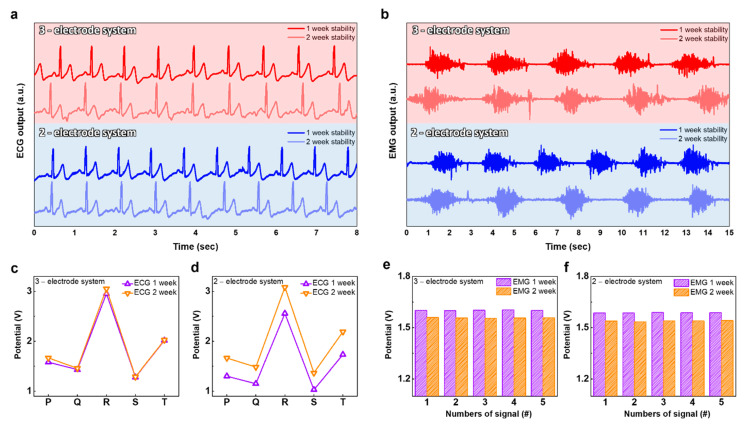
The robustness of the WPD electrodes. (**a**) Repeatability tests for 2 weeks with the WPD electrode for ECG biopotential signals; (**b**) repeatability tests for 2 weeks with the WPD electrode for EMG biopotential signals; the biopotential signal detected for 2 weeks at intervals of 1 week; (**c**) the PQRST peak in 3-electrode system; (**d**) the PQRST peak in 2-electrode system; (**e**) the quantified EMG biopotential signals in 3-electrode system; (**f**) the quantified EMG biopotential signals in 2-electrode system.

Next, we investigated the proposed electrode’s robustness by reusing the same WPD electrode for 2 weeks. The ECG and EMG biopotential signal measurements were performed utilizing reused WPD electrodes at 1-week intervals (Figure 7a,b). The ECG and EMG biopotential signals were clearly detected by the reused WPD electrodes. As a result, the WPD electrode showed superior performance in detecting ECG and EMG biosignals over two weeks. We also plotted the PQRST peaks of the ECG biopotential signal detected with the reused WPD electrodes at 1-week intervals (Figure 7c,d). The ECG biopotential detected by the two-electrode system presented a 101 mV insignificant difference biopotential at the R peak. On the other hand, in the ECG biopotential signals measured with the three-electrode system, the peak intensity differed by an average of 402 mV. The plotted EMG biopotential signal intensity decreased as the number of reuses of the WPD electrode increased (Figure 7e,f). The EMG averaged biopotential signals measured with the two-electrode system and the three-electrode system had a difference of 44 mv and 47 mv, respectively. Again, the biopotential readings were obtained from the sensor node described in Figure 2.

## 4. Conclusions

In summary, we presented waterproof dry-type PEDOT:PSS (WPD) ECG and EMG electrodes protected from external liquid using a double layer of a polyimide (PI) substrate and fluorinated polymer CYTOP. The morphological characteristics and chemical composition ratios of WPD electrodes were investigated through XPS, UPS, SEM and AFM analysis. Additionally, the waterproofing effect of the PI substrate and CYTOP layer was verified using the contact angle analysis. The WPU and EG improved the flexibility and additional conductivity characteristics in the proposed WPD electrodes, respectively. The WPD electrode clearly detected ECG and EMG biopotential signals using the two-electrode system and the three-electrode system. In addition, the WPD electrode and the commercial AgCl-based wet electrodes showed similar performances in detecting ECG and EMG biopotential signals. The proposed WPD electrode verified the robustness of the electrode by detecting ECG and EMG biopotential signals for 2 weeks. Our study can be applied to dry biopotential electrodes research regarding the reliable detection of biosignals in an external humid atmosphere.

## Supplementary Materials

The following supporting information can be downloaded at: https://www.mdpi.com/article/10.3390/polym14132586/s1, Supplementary Figure S1: Sheet resistance of PEDOT:PSS electrode and the proposed WPD electrode. Supplementary Figure S2: The UPS analysis of the PEDOT:PSS. Supplementary Figure S3: Optical bandgap of the PEDOT:PSS electrode and the proposed WPD electrode shown in the Tauc plot method. Supplementary Figure S4: Photography of WPD electrode degradable performance over time immersed in cold water.
