# Supplementary material for: Blended Polymer Dry Electrodes for Reliable Electrocardiogram and Electromyogram Measurements and Their Eco-Friendly Disposal Led by Degradability in Hot Water
Source: Polymers (Basel). 2022 Jun 26;14(13):2586. doi: 10.3390/polym14132586 (PMC9269162; doi:10.3390/polym14132586)
Supplement: Supplementary file 1 [file polymers-14-02586-s001.zip › polymers-1738192-supplementary.pdf]

polymers

MDPI

Supporting Information

Blended Polymer Dry Electrodes for Reliable Electrocardiogram and Electromyogram Measurements and their Eco-Friendly Disposal Led by Degradability in Hot Water

Dong Hyun Lee [1,†], Eun Kwang Lee [2,†], Chae Hyun Kim [1,†], Hyung Joong Yun [3] Young-Joon Kim [1,*], and Hocheon Yoo [1,*]

[1] Department of Electronic Engineering, Gachon University, 1342 Seongnam-daero, Seongnam 13120, Republic of Korea; danny99hodam@gachon.ac.kr, gch427@gachon.ac.kr
[2] Department of Chemical Engineering, Pukyong National University (PKNU); eklee@pknu.ac.kr
[3] Advance Nano Research Group, Korea Basic Science Institute (KBSI), Daejeon 34126, Republic of Korea; hjyun@kbsi.re.kr

† These authors contributed equally to this work
* Correspondence: youngkim@gachon.ac.kr(Y.K.); hyoo@gachon.ac.kr(H.Y.)

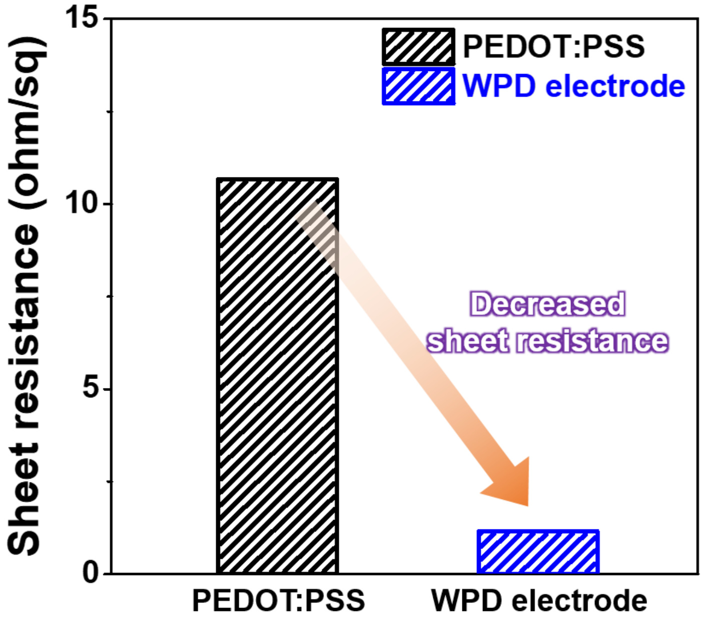

Figure S1 Sheet resistance of PEDOT:PSS electrode and the proposed WPD electrode

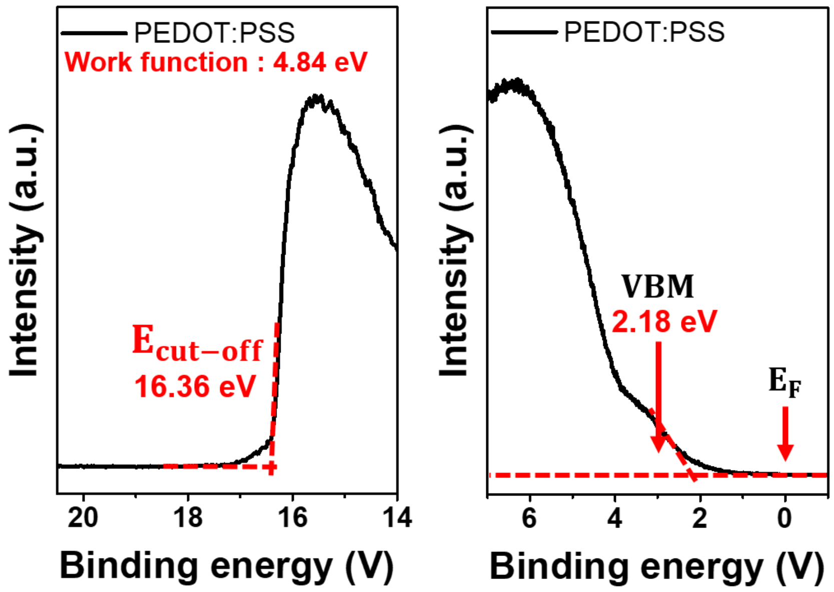

Figure S2 The UPS analysis of the PEDOT:PSS.

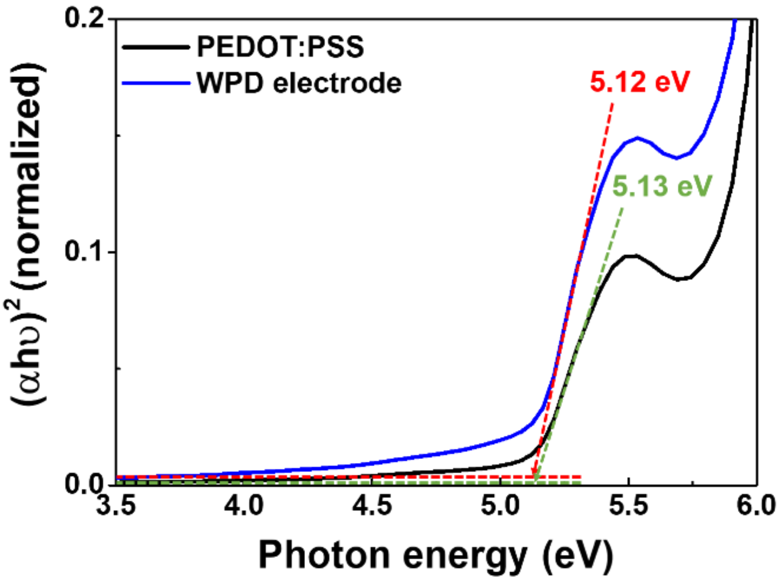

Figure S3 Optical bandgap of the PEDOT:PSS electrode and the proposed WPD electrode shown in the Tauc plot method.

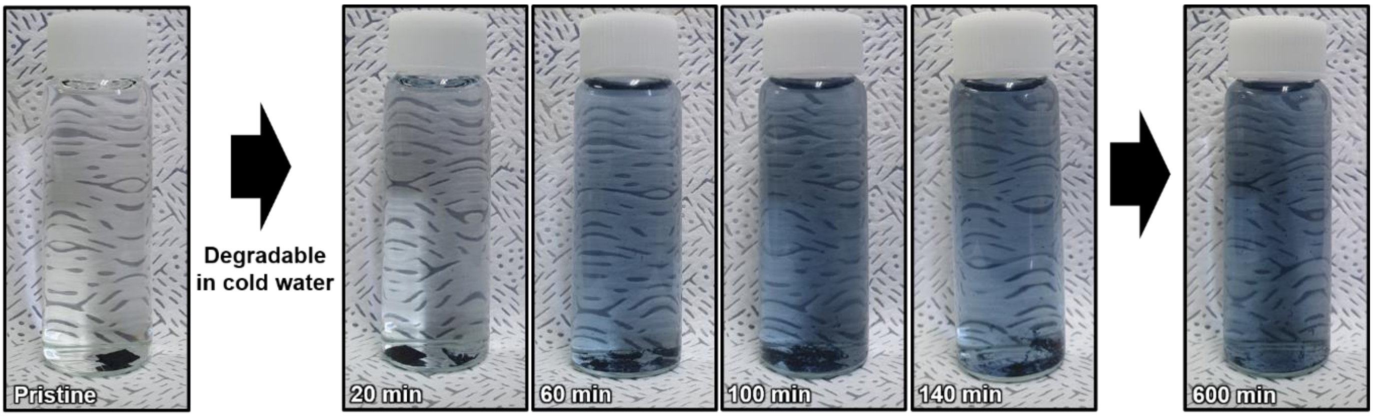

Figure S4 Photography of WPD electrode degradable performance over time immersed in cold water..

.
